# Cyro-EM structure of human mGlus: leading therapeutic potential to neurological diseases

**DOI:** 10.1038/s41392-021-00720-2

**Published:** 2021-08-16

**Authors:** Huan Xiao, Qiu Sun

**Affiliations:** State Key Laboratory of Biotherapy and Cancer Center, West China Hospital, Sichuan University and Collaborative Innovation Center for Biotherapy, Chengdu, China

**Keywords:** Structural biology, Diseases

Recently, Lin et al.^1^ and Du et al.^2^ have published two papers in *Nature*, unveiling the full-length structures of metabotropic glutamate receptors (mGlus) of their homologous and heterodimers, and illustrating their conformational change from inactive to fully activated state, which provides a mechanistic basis for a comprehensive understanding of class C G-protein-coupled receptors (GPCRs).

Glutamate is an important neurotransmitter that mediates synaptic excitability and synaptic transmission in the central nervous system in mammals. It is involved in many important physiological functions such as learning, memory, and neurodevelopment in the brain.^3^ Glutamate receptors include ionotropic glutamate receptors (iGlus) and mGlus. mGlus belong to the class C GPCRs family, which can be divided into 8 different subtypes mGlu1-8. mGlus response to glutamate through a series of cell signaling pathways such as G-protein-coupled signaling pathways, which are important therapeutic targets for neurological diseases such as Alzheimer and schizophrenia. However, no drugs targeting this receptor family have been successfully marketed so far and the regulatory mechanism of mGlus remains unclear, especially how the conformational change of VFT triggers TMDs twisted and activates signal transduction.^4^ It was previously recognized that homo or hetero dimerization is indispensable for the function of mGlus and the complexes are allosteric proteins with two subunits that interact with each other. In the homodimer of mGlus, it is mysterious that only one receptor subunit is responsible for coupling with G protein during receptor activation, whereas in the different types of heterodimers, the function modulations are very complex.^5^ Recent studies from Lin et al. and Du et al. addressed this issue by resolving high-resolution structures of mGlus in different states, providing critical insights into the mGlus regulation, which facilitate the development of related drugs.

**Fig. 1:**
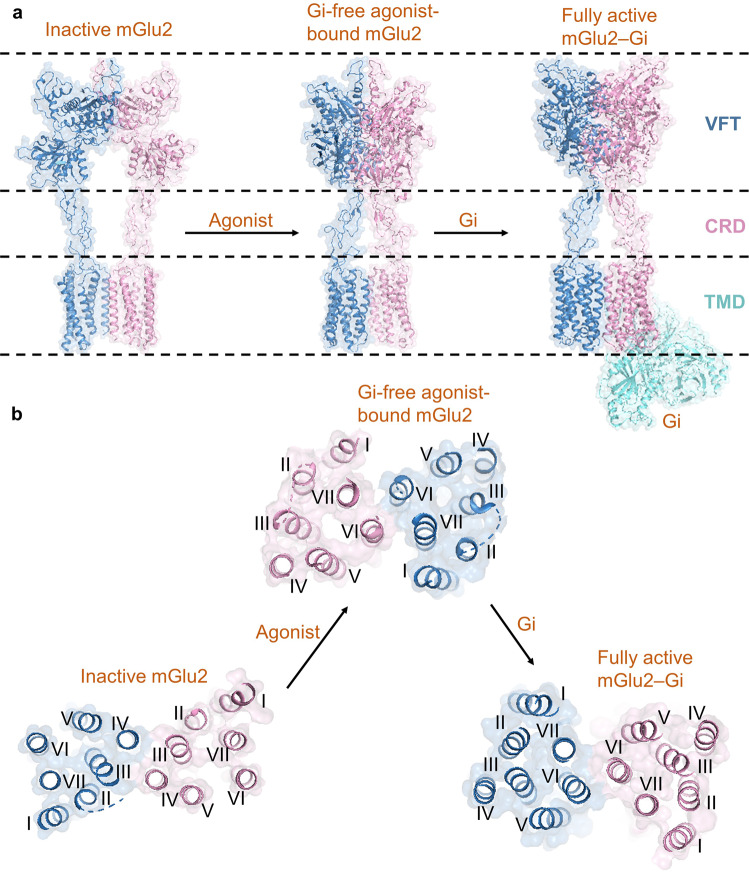
Conformational changes of mGlu2. **a** Cryo-EM structures of the inactive mGlu2, Gi-free agonist-bound mGlu2, and fully active mGlu2–Gi. **b** Dimerization modes of mGlu2 TMD from inactivation to activation

Lin et al. reported the fully active structures of human mGlu2 and mGlu4 bound to heterotrimeric Gi protein, which confirmed asymmetric dimerization is essential to receptor activation (Fig. 1a). After the receptor’s extracellular domain is combined with the agonist, its conformation changes from an open state to a closed state, driving the transmembrane domains (TMDs) to be substantially twisted, and the interface between the two subunits is converted from TM4-TM4 to TM6-TM6 symmetry interface. When the receptor binds to the G protein, the TMDs are further twisted, inducing TM5 and TM6 of one subunit and TM1, TM6 and TM7 of another subunit form an asymmetric dimer interface (Fig. 1b). The receptors form an asymmetric dimer for receptor activation, which affects their internal conformational rearrangement, resulting in only one subunit suitable for binding to Gi proteins. In addition, after the Gi protein binds to one of the subunits, it forms a steric hindrance to prevent the other subunit from binding to the Gi protein. The study provides molecular basis and mechanism insights for the asymmetric signal transduction of mGlus and the Gi protein binding and receptor activation of class C GPCRs.

Du et al. reported four Cryo-EM structures of human mGlu subtypes mGlu2 and mGlu7, including inactive mGlu2 and mGlu7 homodimers; mGlu2 homodimer with an agonist and a positive allosteric modulator (PAM), and an inactive mGlu2-mGlu7 heterodimer. The studies revealed that different mGlu heterodimers are associated with a subtype-dependent dimerization mode of signal transduction. The mGlu7 subunit is more dominant in controlling dimeric association and G-protein activation in heterodimers, as the TMDs in the mGlu2–mGlu7 heterodimer assemble in a manner similar to that in the mGlu7 homodimer which is also supported by functional data. In the mGlu2–mGlu7 heterodimer, the mGlu2 subunit is capable of G-protein coupling when its TMD activation is facilitated by PAM. However, if the mGlu7 blocked subunit was locked in its inactive state with a selective NAM the signal transduction could not be redirected to mGlu2. This is in contrast with previous findings on the mGlu2–mGlu4 heterodimer, where mGlu2 took over for G-protein activation when mGlu4 NAM was used. This is the first report which has provided structural information for the mGlus heterodimerization and laid a solid foundation for further understanding of the molecular regulation mechanism of mGlus heterodimers.

In conclusion, these two studies have analyzed the three-dimensional structure of a variety of human mGlus in different dimerization states through cryo-electron microscopy technology. It fully explained the fine conformational changes of mGlus from inactivated to fully activated state and revealed the complex signal transduction patterns of their homodimers and heterodimers. The mGlus play an irreplaceable role in many neurodevelopment and mental diseases. The studies provide a deeper and more comprehensive understanding of class C GPCRs and provide an insight for in-depth study of the signal transduction mechanism of mGlus in the central nervous system. Such structure-based drug research and development will make a significant breakthrough in the near future.
